# The REThink Online Therapeutic Game: A Usability Study

**DOI:** 10.3390/children10081276

**Published:** 2023-07-25

**Authors:** Ioana Alexandra Iuga, Cristina Teodora Tomoiaga, Oana Alexandra David

**Affiliations:** 1 Evidence-Based Psychological Assessment and Interventions Doctoral School, Babeș-Bolyai University, 400015 Cluj-Napoca, Romania; ioana.iuga@ubbcluj.ro (I.A.I.); cristinalorint@psychology.ro (C.T.T.); 2 Department of Clinical Psychology and Psychotherapy, Babeș-Bolyai University, 400015 Cluj-Napoca, Romania

**Keywords:** adolescents, children, emotional problems, gamification, serious gaming

## Abstract

Background: Children and adolescents’ help-seeking behaviors are often limited by fear, uncertainty, and stigma, as well as challenges with finding the right intervention, help, and a lack of familiarity with the process. A promising direction for the development of therapeutic interventions targeted at children is embedding them into gamified interventions, known as therapeutic or serious games. The aim of this paper is to describe the protocol of the beta REThink therapeutic game and to evaluate the usability of the game in a sample of children and adolescents. Methods: The study was delivered in schools, where 137 children and adolescents aged between 9 and 16 years old played the levels of the REThink game, followed by filling in the usability questionnaire. Findings: The results show above average evaluations for all levels of the game, for every dimension evaluated, namely presence/immersion, enjoyment, learning effectiveness, narratives, goal clarity, adequacy of learning material, and motivation. This study contributes to the literature on the usability of cognitive behavioral therapy-based therapeutic games for improvements in the emotion regulation abilities of children and adolescents, which can guide researchers interested in testing the REThink game in different protocols, as well to encourage its use by mental health specialists and parents.

## 1. Introduction

Results of studies investigating children’s mental health reveal a concerning trend, indicating increased rates of mental illness and a higher prevalence of them. A meta-analysis conducted during the COVID-19 pandemic [1], indicates that the prevalence of anxiety and depression among children and adolescents was 29% and 26%. Additionally, 20.9% of adolescents aged 12–17 from the United States, evaluated between 2018 and 2019, reported ever having a major depressive episode, 36.7% reported persistent feelings of sadness and hopelessness, while 18.8% were seriously considering suicide [2].

Even though they face emotional problems, children and adolescents’ help-seeking behaviors are often limited. There are several factors that can contribute to their tendency to not access mental health services such as fear of what can happen to them during treatment, the need to trust an unknown person (a mental health professional), uncertainty caused by their lack of understanding of mental health problems and/or treatment and what it implies, and the stigma of being judged by their families, as well as challenges with finding the right intervention, help, and the lack of familiarity with the process. Youths identified several factors that could encourage their engagement in mental health interventions [3]. Flexibility in the delivery of intervention was considered an important factor, with services meeting the needs of consumers, improved quality of information, and materials being provided in the intervention emerging as the main themes. Further, personalized care plans and opportunities for children to exercise their independence were found to be of great importance [4]. As youths with mental health difficulties need to benefit from specialized interventions, it is important that the design and the delivery of services captures their experience and offers support across different socio-economic and cultural contexts.

A recent report submitted by the United Nations International Children’s Emergency Fund (UNICEF) shows that 59% of school-aged children from Eastern Europe have internet access at home [5]. In 2014, 6.3% of Romanian children were using a mobile phone, with the most common age for a child to first own a mobile phone being 10 years old [6]. The widespread access to technology in Europe, Romania included, presents a great opportunity for mental health specialists to connect with this population segment.

Internet-based psychological interventions were the first ones to make use of the growing access to the internet in order to improve youths’ mental health outcomes. Studies investigating the effectiveness of internet-based interventions for children and adolescents show promising results concerning anxiety symptoms [7], obsessive compulsive disorders [8], and depression [9]. Several meta-analyses indicate that internet-based interventions are just as effective as ones delivered in person [10,11]. Internet and technology-based interventions can take many forms, keeping up with technological advances.

Cognitive behavioral therapy (CBT) is considered particularly appropriate for online delivery, as it is highly structured and can be implemented in a sequential manner. Consequently, interventions that employ CBT techniques have been implemented using computers [12,13], mobile phones [14], and virtual reality devices [15,16]. Grist [14] summarized the characteristics of existing evidence-based mental health apps, many of them including self-monitoring tools, psychoeducational resources, CBT skills training, and symptom assessment. Although these apps have the advantage of being evidence-based, specialists call for means to increase user engagement within the app.

A promising direction for the development of therapeutic interventions targeted at children is the one of serious or therapeutic gaming, built on gamification principles [17]. There are several theoretical foundations for the development of gamification [18]. The goal-setting theory [19] directs serious game developers towards including specific and appropriately difficult goals within the game, that would support the emergence of flow experience, characterized by a balance between the challenge’s difficulty and the user’s skills [20]. This would warrant the game’s difficulty to be personalized for different levels of users’ ability. Another important factor in serious game development is represented by individual goal setting in order to enhance effort and persistence in activities [18]. Gamification in the context of mental health apps should also provide direct feedback and positive reinforcement for the players, promoting self-efficacy [21]. Allowing users to see their peers’ performance may also enhance user engagement, based on the basic need of conformity and proximity with peers [22].

Several online therapeutic games have been developed and validated in previous years, focusing on the treatment of anxiety [23,24]. Among them, the “MindLight” game uses relaxation techniques and biofeedback to guide players through the game, rewarding players for responding to positive stimuli and disengaging from negative stimuli [25]. The therapeutic game “Dojo” [26] trains youth in navigating negative emotions such as anger, frustration, and fear, while gaining points for their actions.

The online REThink therapeutic game is an online innovative action game, created based on rational emotive behavioral therapy (REBT), intended at preventing emotional problems in children and adolescents. The REThink game can be used in a transdiagnostic manner and has been found to be effective at improving emotion regulation skills, depressive moods, and emotional symptoms in a sample of children aged 10–16 [27].

The REThink therapeutic game as a technology-based intervention has several advantages and unique characteristics. Firstly, it does not require specialized equipment in order to be used. While other similar games might require specialized headsets (e.i., MindLight), REThink can be accessed by youths using devices that are everyday tools for them (mobile phones and laptops), a feature that can increase accessibility, especially for marginalized groups. Another feature that sets apart the REThink game is the wide range of strategies that are included within the levels. In addition to specific strategies that are invoked by other similar apps (bias towards positive stimuli—MindLight; emotion recognition—Dojo), the REThink game offers several other strategies (i.e., problem solving, the identification of rational and irrational beliefs, cognitive change, and mindfulness), that children can adapt and use flexibly in accordance with the specifics of the difficult situation that they may be facing, in a transdiagnostic manner. These features contribute to the efficacy of the REThink game as a standalone action game that incorporates a full protocol for the prevention of emotional difficulties in children.

Rational emotive behavioral therapy [28] and rational emotive behavior education [29] represent a foundation for the REThink game. Based on the aforementioned theories, the mechanism of change in the game is the change in children and adolescents’ beliefs and fostering of psychological resilience through rational beliefs [27] such as frustration tolerance, and unconditional self/other/life acceptance. This is accomplished through educational and experiential learning modules aimed at teaching kids how to recognize their beliefs, reframe their inaccurate beliefs in order to transform their dysfunctional emotional reactions, employ efficient problem-solving and decision-making techniques, and develop good emotions and social skills.

## 2. REThink Game Protocol

The REThink game is a therapeutic video game that can be used as a standalone application, designed to promote emotional resilience in children and adolescents. The game is accessible online through a browser, or via downloading it from the Google Play or Apple App stores in two versions: a full game with seven complete levels, or a trial version with limited access to specific parts of each level. The trial version represents the game’s evaluation system which assesses the skills of the players before they are trained in the game.

**Figure 1:**
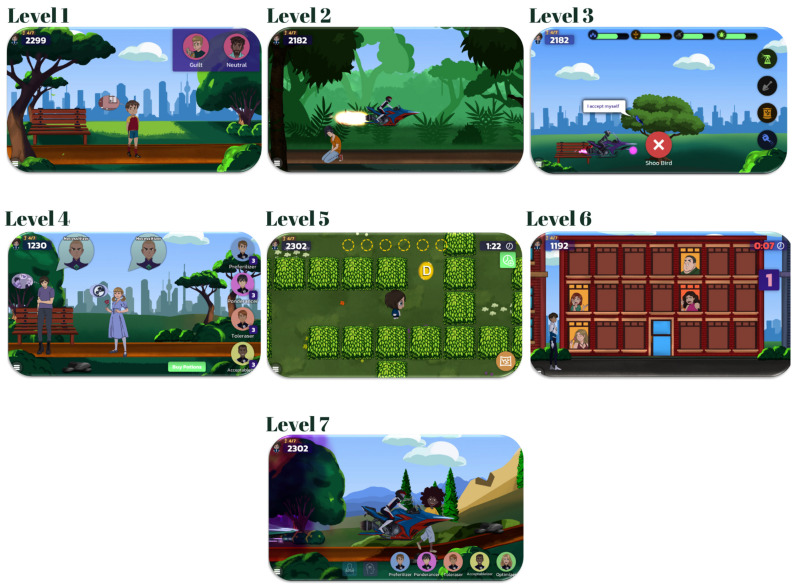
REThink game levels.

The player’s mission is to help the people on Earth escape the power of Irrationalizer, the antagonist, representing an irrational character, due to his capacity of cultivating bad minds and instilling irrational thinking and unhealthy emotions. The game includes the main positive character, RETMAN, and his helpers, five rational characters who are the player’s friends: Preferilizer (representing preference beliefs), Ponderancer (representing non-awfulizing beliefs), Toleraser (representing high frustration tolerance beliefs), Acceptableizer (representing unconditional acceptance beliefs), and Optimizer (representing happiness). Irrationalizer is RETMAN’s enemy, who promotes irrational thinking, together with his servants: Necessitizer (representing demandingness beliefs), Awfulizer (representing awfulizing beliefs), Frustralizer (representing low-frustration-tolerance beliefs), and *Discourager* (representing global evaluation beliefs). The game has seven levels (see Figure 1). Each level has various degrees of complexity, which increase along with the player’s progress in the game, and begins with a questionnaire that measures the targeted skills. The levels conclude with a cognitive, emotional, or behavioral challenge for the player to complete. The game is currently available with an English storyline and subtitles in six languages: Romanian, English, German, Spanish, French, and Italian. Rigorous studies support the game’s effectiveness at reducing depressive moods, improving emotional awareness and control [30], and changing irrational cognitions [27], with maintained changes (e.g., improvements in depressive mood) at 6 months of follow-up [31]. The REThink Game protocol can be consulted in detail in the Supplementary Material.

The main aim of the study is to assess the usability of the REThink game for children and adolescents aged 9 to 16 years old. A secondary objective is to explore possible differences in usability ratings across the genders and school levels of the participants. We expect the game to receive above-average scores for all SGES dimensions for the overall game and individual game levels.

## 3. Method

### 3.1. Setting and Participants

The REThink game is targeted at the prevention of emotional problems in children and adolescents. The age range of the participants we chose from the student population was in line with that of the intended audience. The sample consisted of 330 children and adolescents aged between 9 and 16 years old (*M* = 12.26) from various counties in Romania.

### 3.2. Procedure

Principals of schools in Cluj-Napoca, Romania, as well as the County Center of Resources and Educational Assistance received invitations to collaborate through email. The invitations included information about the research objective, target population, planned activities, and instruments to be utilized by the children. Between September 2021 and June 2023, a series of studies were conducted within the REThink Emotions project, each of them having including an assessment of our instrument’s usability. Parents from the collaborating institutions completed letters of informed consent either handwritten or digitally, using the REThink Emotions platform and app. All information was gathered in class, under the supervision of the REThink team, through the REThink Emotions platform and app. Children had access to several levels of the REThink game, were given sufficient time in order to familiarize themselves with the levels of the game, and then were required to fill out an online usability survey referring to one level of their preference and a demographic questionnaire about their age, gender and grade level.

### 3.3. Measure

The Serious Game Evaluation Scale (SGES) [32] is a self-report measure that assesses user’s experience after interacting with digital educational applications. Specifically, we used items referring to presence/immersion, enjoyment, learning effectiveness, narratives, goal clarity, adequacy of learning material, and motivation. Presence refers to a psychological state in which users experience virtual items as real [33], while immersion is the experience of “being in the game”, where players are unaware of the passage of time and their surroundings [34]. Enjoyment refers to the pleasure and fun people have while playing serious games. Learning effectiveness items inquire about ways in which the game facilitates the user’s knowledge acquisition. The narratives of the game were assessed in terms of the clarity of the storyline and interest in the events presented.

The SGES includes twelve factors and fifty-three items, presented in a 5-point Likert-type scale, ranging from “Strongly Agree”, to “Strongly Disagree”. In this study, we used several subscales from the scale, namely presence (I forgot about time passing while using the game), enjoyment (It felt good to successfully complete the tasks in this game), learning effectiveness (This game was a much easier way to learn compared to the usual teaching), narratives (I was captivated by the game’s story from the beginning), goal clarity (The game’s goals were presented at the beginning of the game), adequacy of the learning material (The good organization of the content helped me to be confident that I would learn this material), and motivation (When using the game, I did not have the impulse to learn more about the learning subject). After completing the game, we allowed the participants to choose which level to evaluate.

The goals of each level were evaluated in terms of specificity and promptness throughout the level. The complexity and level of information understanding were used to determine the suitability of the learning material. Finally, children’s motivation to learn through the use of the serious game was measured. The SGES shows adequate psychometric properties, and the confirmatory factor analysis conducted using the scale showed good fit indices. The scale’s internal consistency was found to be adequate, with a Cronbach’s alpha ranging from 0.88 to 0.95 for the individual subscales and 0.96 for the overall score [30,32]. For the current sample, the Cronbach’s alpha coefficients were adequate, with a coefficient of 0.72 for presence, 0.86 for enjoyment, 0.92 for perceived learning effectiveness, 0.85 for narratives, 0.87 for goal clarity, and 0.75 for motivation, and a lower coefficient of 0.65 for the adequacy of the learning material. SGES is a widely used scale for evaluating usability from different perspectives of serious games [32,35]. The scale was selected based on its psychometric proprieties and because it has been used in other studies to assess the usability of other serious games/digital games.

### 3.4. Data Analysis

We first performed descriptive analysis to examine the scores provided for the game as a whole; then, we looked specifically at each level. Next, multivariate analysis of variance was used to compare usability scores across participants’ school levels (elementary school, lower secondary and upper secondary). Finally, we employed regression analyses in order to determine if gender significantly predicts usability ratings, and *t*-tests were used to identify differences in usability scores between males and females.

## 4. Results

### Sample Characteristics

Sample characteristics are presented in Table 1. Out of the total sample, 57% of the sample was female and 39.7% male, while 3.3% chose not to disclose their gender. The study was delivered in schools, and we recruited 52 elementary school participants, 94 from lower secondary schools, and 93 participants from upper secondary schools, while the rest chose not to disclose their grade level.

Out of the 330 participants in the sample, 73 participants evaluated Level 1, 31 evaluated Level 2, 37 evaluated Level 3, 74 evaluated Level 4, 20 evaluated Level 5, 45 evaluated Level 6, and 50 evaluated Level 7.

The overall results showed above-average evaluations for our game. Results can be seen in detail in Table 2. The analysis showed a mean score of 3.11 (*SD* = 0.85) for the presence subscale, a mean score of 3.16 (*SD* = 0.99) for the enjoyment subscale, and one of 3.15 (*SD* = 1.05) for the learning effectiveness subscale. For the narratives subscale, we obtained a mean score of 3.48 (*SD* = 0.914), for the goal clarity subscale we obtained a mean of 3.24 (*SD* = 1.08), for the adequacy of learning material subscale we obtained a mean of 3.13 (*SD* = 0.81), and for the motivation subscale we obtained a mean of 3.08 (*SD* = 1.00). All obtained means are above-average; overall, these results indicate good usability, with the highest score for the narratives subscale and the lowest score for the motivation subscale. Since the scale does not have norms, the interpretation of the scores is based only on the mean and direction of the scores.

As a next step, we analyzed the usability scores indicated for individual levels, the results showing, again, above-average evaluations. The scores ranged from the highest mean score of 3.46 for the narratives subscale (*SD* = 0.87, *n* = 57) to the lowest mean score of 3.03 (*SD* = 0.74, *n* = 57) for the presence subscale for Level 1. In the case of Level 2, we had between 25 and 31 evaluations, the highest mean being 3.40 for the goal clarity subscale (*SD* = 0.79) and lowest mean being 2.82 (*SD* = 1.01) for the presence subscale.

We had 37 evaluations for Level 3, with the scores ranging from 3.27 for motivation (*SD* = 1.05) to 2.69 (*SD* = 1.06) for the goal clarity subscale, and for Level 4 we had 74 evaluations, with mean scores ranging from 3.53 (*SD* = 0.82) for narratives to 3.157 (*SD* = 0.78) for the presence subscale.

For Level 5, we had the smallest number of evaluations (*n* = 20), with means ranging from 3.67 (*SD* = 0.77) for narratives to 3.13 for enjoyment (*SD* = 1.06). Level 6 had 45 evaluations with means ranging from 3.60 (*SD* = 0.98) for narratives to 2.96 (*SD* = 1.04) for motivation. For the last level, we had 50 evaluations with means ranging from 3.83 for narratives (*SD* = 0.93) to 2.87 for motivation (*SD* = 1.16).

Next, we analyzed the scores by taking into account the school level of the participants. Having participants from multiple school levels, we found it necessary to differentiate between their evaluations for the REThink game, considering the fact that children’s development can vary across the age range included in the study. Since we conducted the studies in schools, we categorized the participants according to their school level and in our study we had participants from elementary school years (grade 3 and 4), lower secondary (grade 5 and 6) and upper secondary (grade 7, 8 and 9). Results from this analysis can be seen in detail in Table 3. We performed MANOVA analysis and, overall, the model was not statistically significant.

Additionally we performed linear regression in order to determine if the age of the participants influenced their rating of the game. The regression model showed significant results for the perceived learning effectiveness subscale with a r^2^ coefficient of 0.035, and *p* = 0.001, which means that age has a significant impact of the evaluation for this specific subscale. Also, we found a significant regression result of r^2^ = 0.049 and *p* = 0.001 for the goal clarity subscale, meaning that older participants gave higher ratings on this subscale, and in the same direction a significant regression resulted for the enjoyment subscale with the results r^2^ = 0.032, *p* = 0.001. The regression model was not significant for presence, narratives, motivation, adequacy of learning material.

We also performed a *t*-test to identify if there were significant differences between boys and girls in terms of their evaluations. Our analysis did not show any significant differences for subscales.

## 5. Discussion

The aim of this paper was to clearly describe the protocol of the REThink therapeutic game and to evaluate the usability of the game in a sample of children and adolescents. Overall, our results show that the REThink game has above-average ratings for all usability dimensions, with the best score for narratives, making it a valid, easy-to-use, and attractive therapeutic instrument for children and adolescents. This is a crucial finding for therapeutic games, since players must be attracted by the storyline in order to better accept the intervention, and comprehend the objectives and requirements of the game in order for it to be successful. The fact that the REThink game is a therapeutic instrument that is attractive, easy-to-understand and to use improves its likelihood of actually being used in schools by counselors, at home by parents, or by practitioners in their private practice as an instrument for the training of emotional abilities in children and adolescents.

Mean scores for the Presence subscale show that the virtual objects presented in Level 7 are perceived as the most realistic by users, while the ones in Level 2 are perceived as the least realistic. The narratives of Level 7 received the highest mean ratings, supporting its quality of fictional background and declarative information provided for users, while Level 3 received the lowest ratings. Level 4 was assessed as having the highest learning effectiveness, facilitating users’ knowledge acquisition, while Level 3 received the lowest score. Level 3 was perceived as being the most rewarding, facilitating children’s motivation to play, while Level 7 was rated as being the least motivating. The complexity of Level 7, being built as a realistic and information-rich learning environment, could hinder children’s motivation to play while facing a more complex task. The learning objectives and materials provided by Level 5 were rated as being the most adequate, while those of Level 7 received the lowest ratings. While Level 5 provides a structured model of problem solving, Level 7 has the purpose of offering children a context in which to practice skills that are provided in previous levels, together with additional skills that can be of a more abstract nature (i.e., compassion). Level 4 was perceived as having the best designed gaming and learning objectives, and Level 3 was perceived as having the lowest goal clarity ratings. Level 1 was evaluated as being the most enjoyable, and Level 3 received the lowest enjoyment scores. This discrepancy might stem from Level 3’s open ending, in which the game becomes progressively more difficult and children have to stop the level when they become frustrated or cannot keep the pace anymore. Nonetheless, the lowest scores for each dimension evaluated are still above-average ratings, indicating that the REThink game is an appropriate tool, meeting the characteristics proposed in the literature for gamified tools [32].

The lowest score for the overall game was obtained for the goal clarity subscale. More specifically, Level 3 scored lowest (2.697) on this dimension, though it was still in the average score range. This level refers to contents that are harder to understand, especially for children, and might require more playtime or additional explanations from a therapist. However, being an interventional tool, it is expected to be played several times in order to obtain the desired results.

Out of all the game levels, Level 4 and Level 7 received the best scores on multiple subscales, namely presence, narratives (Level 7), goal clarity, learning effectiveness and enjoyment (Level 4). These results imply that Level 4 and 7 are the most engaging, clear, and easy-to-understand of all levels.

On the other hand, Level 3 received the lowest scores on the majority of subscales, namely enjoyment, narratives, goal clarity, and learning effectiveness. Even though all evaluations for Level 3 were above-average, the fact that it was rated the lowest might be explained by the small sample size for this level. Furthermore, this might have been due to the fact that Level 3 introduces the concepts of rational and irrational thinking, and teaches their connection to well-being, which, for the majority of participants, can be difficult to understand and learn. The distinction between irrational and rational thinking, though important, is challenging to understand even for adults, and thus it is expected to be perceived as harder by children and adolescents as well.

Even though the multivariate model was not significant for differences in school levels, our regression analysis indicated that age had an influence on the evaluations of the enjoyment, goal clarity and perceived learning effectiveness subscales, meaning that older participants offered higher ratings. These results might suggest that the REThink game is best-accepted by lower and upper secondary students, and that some adjustments in the game (e.g., adapting the presentation of the level goals) might be required in order to use it effectively with elementary children. Concerning gender differences in the game’s usability ratings, the absence of significant differences between girls and boys supports the REThink game’s successful use by the general youth population, with a positive gaming experience, regardless of gender.

The literature examining the usability of an online therapeutic game for children and adolescents is scarce. Overall, our results are in line with those reported in the literature [32,35] with above-average evaluations, but in a student population. We found one study on learning using different methods of which one was a digital game [36]. The authors used SGES subscales of enjoyment, learning effectiveness and motivation to assess the usability of the digital game for high school students aged 13–16 and their results also showed above-average evaluations. Their evaluations were higher than the ones we found, but the game is different. Our game is a therapeutic intervention which although useful can be harder to play and understand than other types of games.

### 5.1. Limitations and Future Research

This study is not without limitations, and first we would like to mention the number of evaluations on some of the levels. Certain levels received fewer evaluations than others, which may have interfered with data analysis and might have led to biased results. Future studies should include similar numbers of evaluations for each level in order to increase the reliability of the results. More specifically, future studies should evaluate each level after it is played by participants in order to have a more reliable image of its usability and have the same participants rating all the levels.

Another limitation can be the fact that participants played the REThink game only once. As previously stated, the evidence-based content of the therapeutic instrument might be perceived, at first, as hard to understand. Future studies should evaluate the usability of the game with multiple playthroughs for the same levels, in order to observe potential changes in evaluations. Being a therapeutic instrument, it is expected to have multiple playthroughs in order to achieve the desired change. Playing multiple times may affect participants’ evaluations of the game, especially for some subscales (e.g., perceived learning effectiveness).

Another limitation of the present study is that, even though the SGES, which was used for evaluation, was developed specifically for this purpose, there are no norms that we can use in order to judge our results.

### 5.2. Scientific and Practical Implications

This study has scientific implications, contributing to the literature on the usability of CBT-based therapeutic games for improvements in the emotion regulation abilities of children and adolescents. Moreover, it offers important data regarding the therapeutic instrument’s usability, that can guide researchers interested in testing the REThink game in different protocols.

From the practical point of view, this study offers mental health specialists and parents important results and conclusions about youths’ perception and acceptability of these interventions, thus facilitating the use of the game in schools (by counselors) or at home (by parents). According to the results of our study, children and adolescents are fond of and accept the REThink game as a useful intervention tool. The game earned assessments that were above-average, indicating that it has potential as a useful tool for practitioners looking to expand their intervention techniques by including a game-based intervention particularly created to improve emotion regulation abilities. Positive reviews of the game indicate that it may serve as a useful and interesting supplement to conventional therapy methods, strengthening current approaches while potentially improving their results. Due to its portability and accessibility as a smartphone app, parents may utilize it at home with ease. In a familiar and comfortable setting, parents may use the game to teach and improve their children’s emotional regulation skills.

## 6. Conclusions

While specialists and researchers alike search for innovative ways to make use of the numerous technological advancements available, it remains important to consider the acceptance of these tools in the target population, as well as to provide evidence-based support for the use of CBT-based online interventions such as serious games. The present study provides evidence-based support for the use of the REThink game in both research and in practice, being perceived by the users as an enjoyable, immersive, motivating, and efficient therapeutic tool.

## Figures and Tables

**Table 1:** Descriptive analysis for the used sample.

***N***	**Mean Age (*SD*)**	**Gender**			**School Level**			
		**Males *n* (%)**	**Females *n* (%)**	**Not Disclosed *n* (%)**	**Elementary** ***n* (%)**	**Lower Secondary** ***n* (%)**	**Upper** ***n* (%)**	**Not Disclosed** ***n* (%)**
330	12.26 (1.57)	131 (39.7%)	188 (57%)	11 (3.3%)	52 (15.8%)	94 (28.5%)	93 (28.2%)	91 (27.6%)

**Table 2:** Scores on each subscale of SGES and level of the REThink game.

		Presence	Narratives	Perceived Learning Effectiveness	Motivation	Adequacy of Learning Material	Goal Clarity	Enjoyment
Level 1 (*n* = 73)	Mean	3.03	3.46	3.24	3.17	3.22	3.30	3.29
	SD	0.74	0.87	0.99	0.90	0.74	0.99	0.93
	Median	3	3.5	3	3	3	3.33	3.17
	Min; Max	2; 5	2; 5	1; 5	1; 5	2; 5	1; 5	1; 5
	Kurtosis	0.298	−0.612	−0.470	0.181	−0.249	0.95	−0.215
	Skewness	0.157	−0.042	−0.179	−0.110	0.386	−0.504	−0.139
Level 2 (*n* = 31)	Mean	2.82	3.17	3.02	2.95	3.10	3.40	3.18
	SD	1.01	1.02	1.02	0.97	0.72	0.79	1.02
	Median	3	3.25	3.17	3	3	3.33	3.33
	Min; Max	1; 5	1; 5	1; 5	1; 5	2; 5	2; 5	1; 5
	Kurtosis	−0.995	0.716	−0.158	0.027	0.545	−0.163	−0.464
	Skewness	0.009	−0.798	−0.780	0.041	−0.092	0.133	−0.362
Level 3 (*n* = 37)	Mean	2.96	2.96	2.86	3.27	3.11	2.69	2.91
	SD	0.85	0.86	1.05	1.05	0.62	1.06	0.83
	Median	3.13	3	3	3	3	2.67	3
	Min; Max	2; 5	1; 5	1; 5	1; 5	2; 5	1; 5	1; 5
	Kurtosis	−0.112	1.84	0.015	−0.308	0.008	−0.319	−0.379
	Skewness	−0.099	0.005	−0.021	0.200	0.197	0.202	0.106
Level 4 (*n* = 74)	Mean	3.15	3.53	3.27	3.17	3.21	3.41	3.20
	SD	0.78	0.82	0.95	0.973	0.82	0.95	0.78
	Median	3	3.25	3.08	3	3.25	3.33	3.25
	Min; Max	2; 5	2; 5	1; 5	1; 5	1; 5	1; 5	1; 5
	Kurtosis	−0.264	−0.470	−0.266	0.157	0.322	0.316	−0.183
	Skewness	0.464	0.256	−0.231	−0.075	−0.188	−0.448	−0.554
Level 5 (*n* = 20)	Mean	3.19	3.67	3.23	3.17	3.31	3.27	3.13
	SD	0.40	0.77	1.01	0.88	0.95	1.04	1.06
	Median	3.13	3.63	3.25	3	3.25	3.17	3.25
	Min; Max	3; 4	3; 5	1; 5	2; 5	1; 5	1; 5	1; 5
	Kurtosis	−0.002	−0.624	−0.272	0.190	1.28	−0.401	−1.061
	Skewness	0.805	0.698	−0.017	0.639	−0.031	0.149	−0.104
Level 6 (*n* = 45)	Mean	3.21	3.60	3.06	2.96	3.04	3.10	3.11
	SD	0.89	0.98	1.11	1.04	0.94	1.30	1.04
	Median	3.25	3.88	3.17	2.67	3.25	3	3.33
	Min; Max	1; 5	2; 5	1; 5	1; 5	1; 5	1; 5	1; 5
	Kurtosis	−0.209	−1.11	−0.761	−0.602	−0.561	−1.07	−0.499
	Skewness	0.043	−0.195	−0.316	−0.014	−0.451	−0.225	−0.166
Level 7 (*n* = 50)	Mean	3.38	3.83	3.21	2.87	2.89	3.33	3.14
	SD	1.06	0.93	1.23	1.16	0.87	1.28	1.12
	Median	3.5	4	3.25	2.83	3	3.67	3.25
	Min; Max	1; 5	1; 5	1; 5	1; 5	1; 5	1; 5	1; 5
	Kurtosis	−0.539	0.587	−0.816	−0.739	−0.172	−0.941	−0.509
	Skewness	−0.328	−0.791	−0.369	0.28	−0.002	−0.457	−0.540
Total (*n* = 330)	Mean	3.11	3.48	3.15	3.08	3.13	3.24	3.16
	SD	0.85	0.91	1.05	1.00	0.81	1.08	0.85
	Median	3	3.5	3.08	3	3	3.33	3.17
	Min; Max	1; 5	1; 5	1; 5	1; 5	1; 5	1; 5	1; 5
	Kurtosis	−0.209	−0.159	−0.468	−0.227	0.127	−0.464	−0.443
	Skewness	0.068	−0.204	−0.281	−0.025	−0.098	−0.339	−0.307

Note. Means, standard deviation (*SD*) and sample size (*n*) for all outcomes.

**Table 3:** Descriptive statistics for each subscale for grade levels of the participants.

Subscale/School Level	Elementary (*n* = 52)					
	*M*	*SD*	Median	Min; Max	Skewness	Kurtosis
Presence	2.70	0.90	2.75	1; 4	−0.002	−0.473
Enjoyment	2.51	1.18	2.33	1; 5	0.503	−0.654
Learn_effectiveness	2.50	1.15	2.42	1; 5	0.482	−0.527
Narratives	3.11	1.38	2.88	1; 5	0.094	−1.06
Goalclarity	2.55	1.21	2.33	1; 5	0.487	−0.868
Adequacy_Material	2.93	0.94	3	1; 5	0.074	−0.504
Motivation	3.04	1.15	3	1; 5	−0.191	−0.777
**Subscale/School Level**	**Lower Secondary (*n* = 94)**					
	***M***	***SD***	**Median**	**Min; Max**	**Skewness**	**Kurtosis**
Presence	3.22	0.88	3.25	2; 5	0.161	−0.431
Enjoyment	3.31	0.96	3.33	1; 5	−0.219	−0.261
Learn_effectiveness	3.27	0.97	3	1; 5	−0.017	−0.218
Narratives	3.57	0.97	3.5	2; 5	0.200	−0.468
Goalclarity	3.15	0.99	3	1; 5	−0.105	−0.301
Adequacy_Material	3.19	0.81	3	1; 5	0.176	0.132
Motivation	3.20	1.02	3	1; 5	0.152	−0.417
**Subscale/School Level**	**Upper (*n* = 93)**					
	***M***	***SD***	**Median**	**Min; Max**	**Skewness**	**Kurtosis**
Presence	3.00	0.85	3	1; 5	−0.084	−0.231
Enjoyment	3.27	0.86	3.33	1; 5	−0.252	−0.614
Learn_effectiveness	3.24	1.03	3.33	1; 5	−0.468	−0.713
Narratives	3.42	0.91	3.5	1; 5	−0.133	−0.514
Goalclarity	3.46	0.97	3.67	1; 5	−0.736	0.764
Adequacy_Material	3.19	0.80	3.25	1; 5	−0.532	0.772
Motivation	3.11	0.91	3	1; 5	0.032	0.124

Note. Mean (*M*), standard deviation (*SD*) and sample size (*n*).

## Data Availability

The datasets generated and/or analyzed during the current study are not publicly available but are available from the corresponding author on reasonable request.

## Supplementary Materials

The following supporting information can be downloaded at: https://www.mdpi.com/article/10.3390/children10081276/s1, Supplementary Material: Description of the REThink game protocol.
