# Supplementary material for: The REThink Online Therapeutic Game: A Usability Study
Source: Children (Basel). 2023 Jul 25;10(8):1276. doi: 10.3390/children10081276 (PMC10453373; doi:10.3390/children10081276)
Supplement: Supplementary file 1 [file children-10-01276-s001.zip › children-2502064-supplementary.pdf]

Supplementary material: description of the REThink game protocol

Level 1 is designed to help the player in differentiating between the three categories of emotions (positive, negative, and neutral) as well as between emotions that are functional and those that are dysfunctional (e.g., depression vs. sadness; anxiety vs. fear). The player must distinguish between the emotions of the characters and a neutral state shown on the screen (sublevel 1), followed by choosing from 4 basic emotions presented on the screen, and discerning functional or dysfunctional emotions. The trial version of level 1 evaluates the player's ability to identify emotions compared to neutral states.

Level 2 aims to familiarize the player with the relaxation techniques and mindfulness processes. The player's objective is to learn and practice a breathing relaxation technique (sublevel 1) and to develop his/her abilities to be present and mindful, by observing the environment he/she passes through and identifying the elements from the environment (sublevels 2 and 3). The trial version of level evaluates the player's ability to be present.

Level 3 seeks to familiarize the player with the cognitive processes, such as irrational beliefs and rational beliefs. The level is continuous, with three successive sequences: (1) taking care of the tree using purchased resources and removing Irrationalizer's servants who harm the tree (sublevel 1), (2) the appearance of birds (sublevel 2) with rational (should be kept) and irrational thoughts (should be removed), and (3) the rhythm of the game increases, becoming more alert, in order to assess the players' frustration tolerance (sublevel 3). The trial version of level 3 evaluates the player's ability to identify rational and irrational beliefs, and their level of frustration tolerance.

Level four is designed to assist the player in the process of changing irrational cognitions into rational ones. The player is responsible for recognizing the non-player characters' irrational thoughts and provide them with a potion containing the rational version of the thoughts. The player can purchase rational thought potions to alter irrational thoughts. The trial version of level 4 evaluates the player's ability to modify irrational beliefs by using rational alternatives.

Level 5 aims to help the player develop problem solving skills. The action takes place in a maze, where the player must discover the acronym "DECODE", whose letters each represent a problem-solving step: (1) define the problem, (2) explore solutions, (3) compile list, pick solutions, (4) organize a plan, (5) do it, (6) evaluate success. To get out of the maze, the player must put into practice each step of the problem-solving process, which is explained to them when finding the letter corresponding to each step. The trial version of level 5 evaluates the participant's ability to to solve problems quickly in order to exit the labyrinth in the shortest possible time, whilst also collecting the "DECODE" letters.

Level 6 aims to help the player develop attentional skills towards positive stimuli, correcting the attentional bias towards negative stimuli. The action takes place in a town, in front of several residential buildings (that become taller and more populated throughout the course of the level). The player has to quickly identify the happy face out of several faces presenting negative emotions, seen from the windows of buildings. The trial version of level 6 evaluates players' attentional bias.

Level 7 develops the player's ability to make cognitive changes. The player is on a hoverbike and passes by a series of characters who have irrational thoughts that he must change by using rational potions. If the player passes happy characters, they must hug the happy character using the Optimizer character. The player is followed by the servants of Irrationalizer, and if he/she makes a mistake, they catch him. If the servants of the Irrationalizer catch the player, they become affected by irrational thoughts that they have to change. The player has self-compassion resources (choose self-compassion phrases that help him/her, from

6 options) and mindfulness (similar level 2) resources. The level also trains the player's level of compassion. From time to time, randomly, the players can help certain characters. If they help the characters, they either can gain or lose half of the score gained up to that moment. Level 7 trial includes a portion of the complete level.
